# Reattachment of the flexor and extensor tendons at the epicondyle in elbow instability: a biomechanical comparison of techniques

**DOI:** 10.1186/s12891-018-2341-y

**Published:** 2018-12-03

**Authors:** Andreas Lenich, Christian Pfeifer, Philipp Proier, Roman Fleer, Coen Wijdicks, Martina Roth, Frank Martetschläger, Jonas Pogorzelski

**Affiliations:** 1 Department of Orthopedic Sports Medicine, Technical University of Munich, Klinikum rechts der Isar, Ismaninger Str. 22, 81675 Munich, Germany; 2 Helios Clinic Munich West, Department of Orthopedic Sports Medicine, Trauma Surgery and Hand Surgery, Steinerweg 5, 81241 Munich, Germany; 3 German Center for Shoulder Surgery, ATOS Clinic Munich, Effnerstraße 38, 81925 Munich, Germany; 4 Regensburg University Medical Center, Department of Trauma Surgery, Franz-Josef-Strauß-Allee 11, 93053 Regensburg, Germany; 5 Department of Research & Development, Arthrex GmbH, Munich, Germany

**Keywords:** Elbow, Dislocation, Reattachment, Common extensor muscle origin, Common flexor muscle origin

## Abstract

**Background:**

Elbow dislocation represents a common injury, especially in the younger population. If treated surgically, the reattached tendons require a high amount of primary stability to allow for an early rehabilitation to avoid postoperative stiffness. The purpose of this study was to assess the biomechanical properties of a single and a double row technique for reattachment of the common extensor and common flexor muscles origin. We hypothesized that the double row technique would provide greater stability in terms of pullout forces than the single row technique.

**Methods:**

Twelve cadaveric specimens were randomized into two groups of fixation methods for the common extensor tendon or the common flexor tendon at the elbow (1): a single row technique using two knotted 3.0 mm suture anchors, and (2) a double row technique using an additional knotless 3.5 mm anchor. The repairs were cyclically loaded over 500 cycles at 1 Hz from 10 N to a maximum of 100 N (extensors) or 150 N (flexors), and then pulled to failure. Stiffness and maximum load at failure and mode of failure were recorded and calculated.

**Results:**

No significant differences in stiffness were observed between the two techniques for both the extensor and flexor reattachment (*P* = 0.701 and *P* = 0.306, respectively). The mean maximum load at failure indicated that the double row construct was significantly stronger than the single row construct. This was found to be true for both the extensor and flexor reattachment (213.6; SD 78.7 N versus 384.1; SD 105.6 N, *P* = 0.010 and 203.7; SD 65.8 N versus 318.0; SD 64.6 N, *P* = 0.013, respectively).

**Conclusions:**

The double row technique provides significant greater stability to the reattached common flexor or extensor origin to the medial or lateral epicondyle. Thus, it should be considered in the development of improved repair techniques for stabilizers of the elbow.

**Study design:**

Controlled laboratory study.

## Background

The elbow is the most commonly dislocated joint in children and the second most dislocated joint in adults with an estimated incidence of elbow dislocations in the general United States population of about 5.21 per 100,000 person-years [1, 2]. The stabilizing structures of the elbow joint are typically classified as primary, secondary, and dynamic stabilizers [3, 4]. More precisely, primary stabilizers include the bony ulno-humeral articulation, the lateral collateral ligament (LCL) complex as well as the medial collateral ligament (MCL) complex. Secondary stabilizers include the radial head, the anterior and posterior joint capsule, and the common flexor and extensor muscle origins. Finally, the biceps muscle, the brachialis muscle, the anconeus muscle, and the triceps muscle are classified as dynamic stabilizers [3, 4].

While simple elbow dislocations – defined as acute dislocations without concomitant significant fractures - may be accessible through non-operative treatment, complex dislocations involving fractures of the radial head or neck, olecranon, coronoid, humeral condyles or epicondyles typically require surgical intervention [5, 6]. Even though the majority of dislocations can be considered “simple”, “complex” cases still occur in up to 20% of patients suffering from a traumatic elbow dislocation [7]. As the postoperative results have been historically hampered by frequent stiffness with or without recurrent instability, discussions have been raised, whether to augment to the reduction with some type of external fixation or whether the length of postoperative immobilization should be prolonged [8, 9]. However, there is actual consensus in the literature that early rehabilitation following simple elbow dislocation is the best way to prevent range of motion deficits [10]. Whether an early postoperative rehabilitation is safe for the reconstructed certainly depends on the stability of the refixated structures.

Therefore, the objective of this study was to assess the biomechanical properties of a single and a double row technique for re-fixation of the common extensor muscles and common flexor muscles origin. We hypothesized that the double row technique would provide greater stability in terms of pullout strengths than the single row technique.

## Methods

### Specimen preparation

**Fig. 1:**
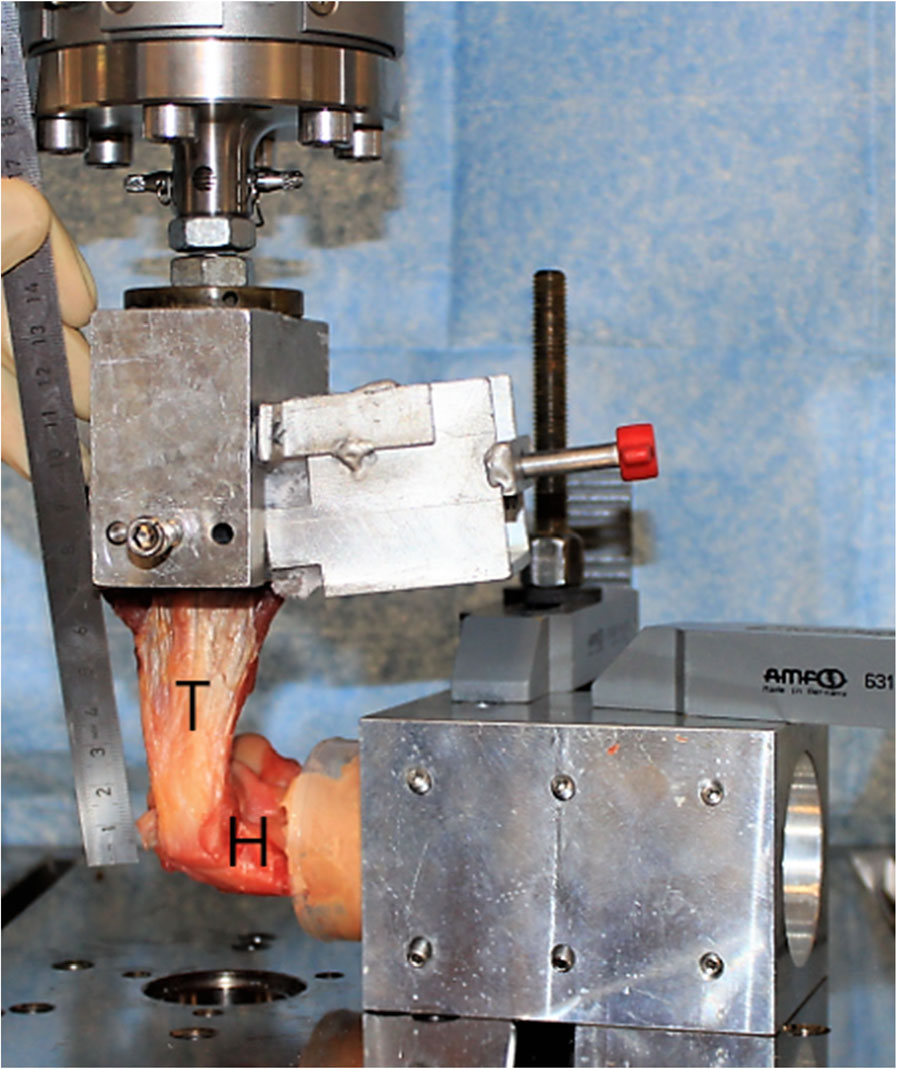
Before testing, care was taken to ensure that the common tendon was aligned vertically to the humeral shaft axis. The distance from tendon insertion to the clamped and frozen muscles was routinely chosen to be 7 cm, while the distance between the most distal part of the humerus and the potted plaster was routinely about 5 cm (T = tendons, H = humerus)

As a cadaveric study, our institution does not require Institutional Review Board (IRB) approval. The study was performed using 12 fresh-frozen, human cadaveric humeri of male donors only, which were donated to our research laboratory. Radial head compression tests were performed to exclude specimens with osteoporosis. More precisely, as mechanical stability of the radial head is known to correlate with bone quality, static axial compression load was applied on the cartilage surface until breakage with a speed of 10 mm/min and subsequently a load-over-displacement analysis performed [11, 12]. To ensure equal bone quality before testing, all specimen with significant deviations in the mean load-over-displacement curve were discarded. All specimens were less than 65 years of age (mean, 55.6 years; standard deviation (SD) 12.0 years), with no history of elbow injury, surgery, or anatomic abnormality and randomized into one of the two groups. Specimens were stored at − 20 °C and thawed at room temperature for 24 h before preparation. The humerus was disarticulated from the ulna and radial bone, and all soft tissue (including the collateral ligaments) except the common flexor origin (consisting of the flexor carpi radialis muscle, the flexor carpi ulnaris muscle, the palmaris longus muscle and the flexor digitorum superficialis muscle) and common extensor origin (consisting of the extensor carpi ulnaris muscle, the extensor carpi radialis brevis muscle, the extensor digitorum muscle, and the extensor digiti minimi muscle) was removed. The humerus was then potted in plaster (Moldasynth, Heraeus Kulzer GmbH, Hanau, Germany) to preserve the position during testing. Care was taken to keep an exact distance of 5 cm from the plaster to the most distal point of the humerus for each specimen (Fig. 1).

### Surgical technique

Two orthopaedic surgeons (Philipp Proier and Andreas Lenich) performed all re-fixations of the extensors and flexors. Two different techniques were used to test fixation strength using a single row or double row technique. Prior to the placing of the anchors, the common flexors and extensors including the cortical bone of its origins were removed.

**Fig. 2:**
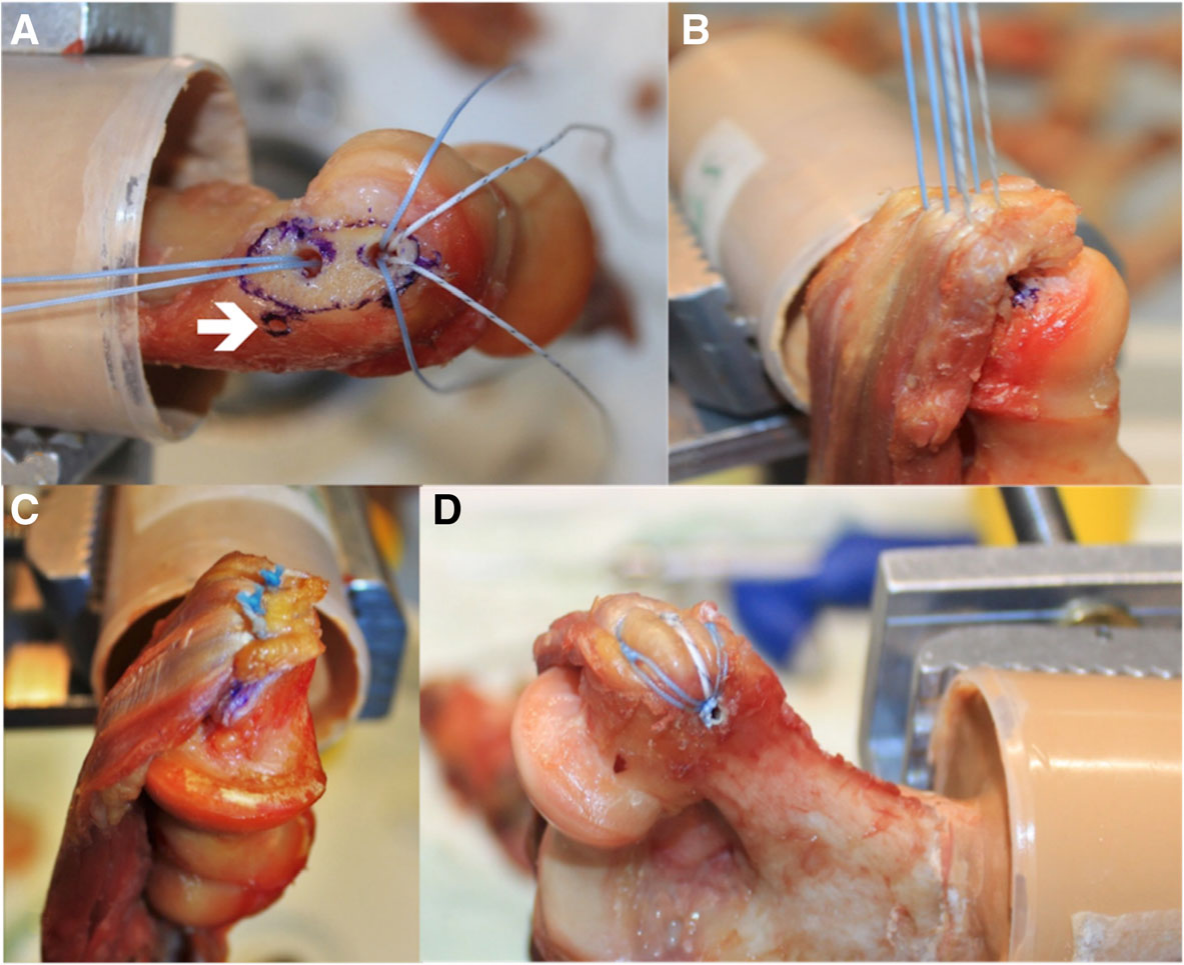
Surgical technique: **a** One single- and one double-loaded suture anchor were placed for the single-row technique in the extended axis of the humeral shaft. If needed, a third anchor was placed posteriorly for the double-row construct (white arrow). **b** All suture limbs of the single-row anchors were shuttled through the tendon in a mattress configuration. **c** Final single-row construct. **d** Final double-row construct

The single row construct (Fig. 2) consisted of one single- and one double-loaded 3.0 mm suture anchor (SutureTak, Arthrex, Inc., Naples, FL) with 2–0 fiber wires to secure the common tendons origin to the medial or lateral humeral bone. With the use of a suitable drill, two holes were positioned like follows: The double-loaded anchor was routinely placed 1 cm proximal to the cartilage-bone-border in the extended axis of the humeral shaft with the same distance to the anterior and posterior joint surface. The single-loaded anchor was subsequently placed 1 cm proximal to the first anchor in the same axis. The six suture limbs of both the double-loaded and single-loaded anchor were shuttled through the common tendon in a mattress technique leaving an approximately 1 cm gap from the tendons margin. Finally, each pair of suture limbs was tied down using seven alternating half hitches and the sutures were cut.

The double row construct (Fig. 1) was similar to the single row construct with the only difference that all six suture limbs of both previously positioned 3.0 mm suture anchors were loaded into the eyelet of a 3.5 mm knotless suture anchor (SwiveLock, Arthrex, Inc., Naples, FL) after tying the knots. Subsequently, a bone socket was created with a punch 1 cm posterior and 0.5 cm proximal to the most proximal anchor of the single row anchors. The eyelet of the anchor was brought to the edge of the socket and the limbs of sutures were individually tensioned. The eyelet was then advanced into the socket until the anchor body contacted the bone, effectively tensioning the suture limbs. Once the anatomy of the common extensor or flexor footprint was restored, the body of the anchor was advanced clockwise into the bone socket to secure the sutures.

### Biomechanical testing

Each construct was biomechanically assed using a dynamic tensile testing machine (Instron ElectroPuls E10000, Instron Systems, Norwood, MA). Before clamping the muscles in a custom fixture approximately 7 cm from the common tendons margin (Fig. 2), the clamps were treated with dry ice to prevent muscle slippage within the fixture during testing. The embedded humerus was securely fixed to the stationary base of the tensile testing machine. After preloading the muscles to 10 N (extensors) or 15 N (flexors), care was taken to ensure that the common tendon was aligned vertically (Fig. 2). The preload of the muscles was defined to be 10% of the natural load of the common extensors or flexors which has been described to be about 100 N for the extensors and 150 N for the flexors [13]. Each construct was cyclically loaded at 1 Hz in 10 steps for five minutes each. The baseline load was 15 N for the extensors and 10 N for the flexors with a stepwise 15 N (extensors) or 10 N (flexors) increase after each step. If the construct was still intact after the 10 steps of cyclic loading, it was pulled to failure at 60 mm/min. Failure was defined as suture breakage or any perceived movement of the implanted anchors. Failure mode was observed and defined in each case by two reviewers. Stiffness as well as maximum load during the pull to failure and mode of failure were recorded and calculated. Stiffness of the repair was calculated as the slope of the load-versus-displacement curve at pull-to-failure (PTF) or the final cycle of cyclic loading if PTF was not reached.

### Statistical analysis

An a priori power calculation was conducted and the usage of six specimens per group was found to be sufficient to detect an effect size of d = 1.2 with 80% statistical power. All continuous variables were not observed to be skewed or over dispersed, so parametric testing methods were used. Thus, t-test models were built to compare the two groups. All statistical analyses and graphics were produced using the statistical program SigmaPlot, version 13.0 (Systat, San Jose, CA).

## Results

**Fig. 3:**
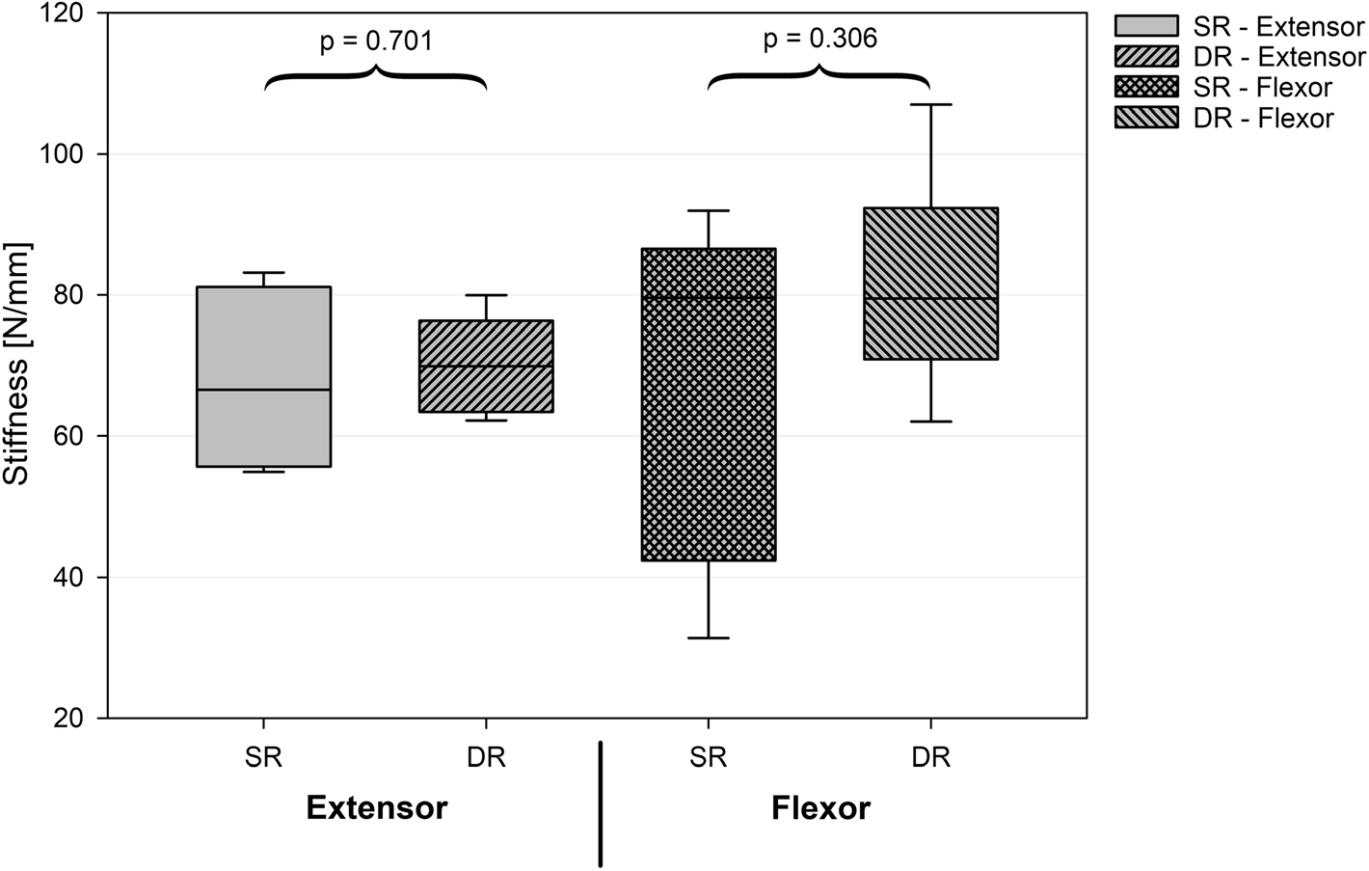
No significant differences in stiffness could be detected between techniques and location of fixation. SR = single row technique. DR = double row technique

**Fig. 4:**
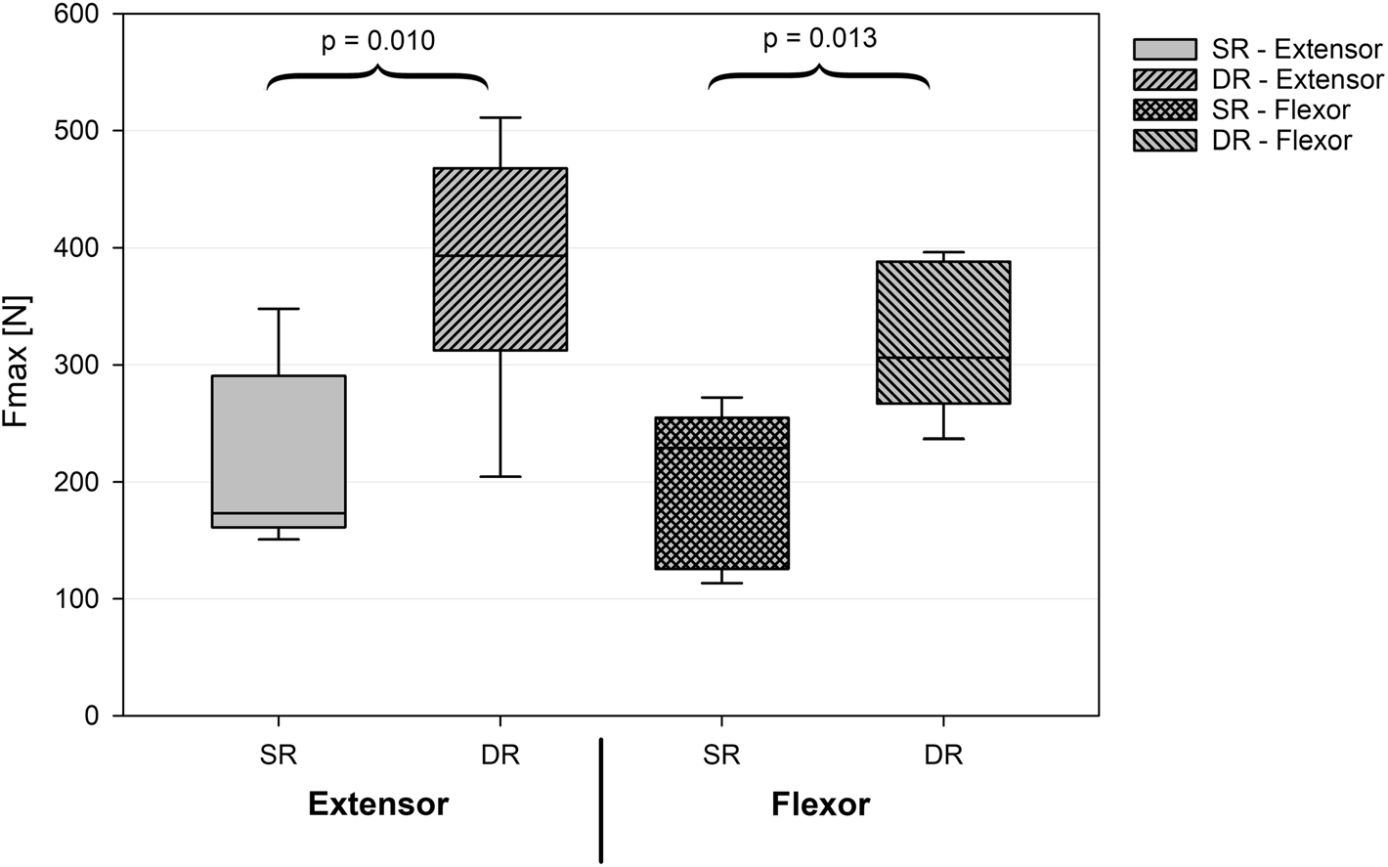
The mean load to failure was significantly higher for the DR technique compared to the SR technique for both extensor and flexor refixation. SR = single row technique. DR = double row technique. Fmax = maximum force

No significant differences in stiffness were observed between the two techniques for both the extensor and flexor refixation (*P* = 0.701 and *P* = 0.306, respectively; Fig. 3). The mean maximum load at failure indicated that the DR construct was significantly stronger than the SR construct. This was found to be true for both the extensor and flexor re-fixation (213.6; SD 78.7 N versus 384.1; SD 105.6 N, *P* = 0.01 and 203.7; SD 65.8 N versus 318.0; SD 64.6 N, *P* = 0.013, respectively; Fig. 4). Two constructs of the SR technique failed before reaching the pull-to-failure testing during cyclic loading while testing the pullout strength of the common flexor refixation (Table 3). None of the DR specimens failed during this phase.

The most commonly observed failure mode for both the SR and DR construct was suture cut out through the tendon. More detailed information of the results of the testing of each technique is given in Tables 1, 2, 3 and 4.

**Table 1:** Overview of the results for testing the SR technique for re-fixation of the common extensor origin

Single Row Technique Extensor	Cycles to Failure	Stiffness of the repair (N/mm)	Maximum load (N)	Pull-to-failure	Failure Mode
Specimen 1	PTF	55.9	171.1	Yes	suture cut out through tendon
Specimen 2	PTF	54.9	150.8	Yes	suture cut out through tendon
Specimen 3	PTF	68.0	175.5	Yes	suture cut out through anchor
Specimen 4	PTF	80.5	348.0	Yes	suture cut out through anchor
Specimen 5	PTF	83.2	164.6	Yes	suture cut out through tendon
Specimen 6	PTF	65.2	271.4	Yes	suture cut out through tendon
Average ± SD		67.9 ± 11.9	213.6 ± 78.7		

Stiffness, maximum load pull-to-failure, and failure mode are presented for each specimen individually. PTF, pull-to-failure; Y = yes, N = Newton, mm = millimeter

**Table 2:** Overview of the results for testing the DR technique for re-fixation of the common extensor origin

Double Row Technique Extensor	Cycles to Failure	Stiffness of the repair (N/mm)	Maximum load (N)	Pull-to-failure	Failure Mode
Specimen 1	PTF	73.3	511.2	Yes	anchor breakage
Specimen 2	PTF	62.2	348.2	Yes	suture cut out through tendon
Specimen 3	PTF	66.6	415.9	Yes	anchor breakage
Specimen 4	PTF	75.1	371.1	Yes	anchor cut out
Specimen 5	PTF	63.9	204.5	Yes	suture cut out through tendon
Specimen 6	PTF	80.0	453.8	Yes	suture cut out through tendon
Average ± SD		70.2 ± 7.0	384.1 ± 105.6		

Stiffness, maximum load pull-to-failure, and failure mode are presented for each specimen individually. PTF, pull-to-failure; Y = yes, N = Newton, mm = millimeter

**Table 3:** Overview of the results for testing the SR technique for re-fixation of the common flexor origin

Single Row Technique Flexor	Cycles to Failure	Stiffness of the repair (N/mm)	Maximum load (N)	Pull-to-failure	Failure Mode
Specimen 1	352	31.4	113.3	No	suture cut out through tendon
Specimen 2	PTF	84.8	248.8	Yes	suture cut out through tendon
Specimen 3	402	46.0	129.6	No	suture cut out through tendon
Specimen 4	PTF	92.0	232.6	Yes	suture cut out through tendon
Specimen 5	PTF	80.5	225.7	Yes	suture cut out through tendon
Specimen 6	PTF	78.7	272.0	Yes	suture cut out through tendon
Average ± SD		68.9 ± 9.9	203.7 ± 65.8		

Stiffness, maximum load pull-to-failure, and failure mode are presented for each specimen individually. PTF, pull-to-failure; Y = yes, N = Newton, mm = millimeter

**Table 4:** Overview of the results for testing the DR technique for re-fixation of the common flexor origin

Double Row Technique Flexor	Cycles to Failure	Stiffness of the repair (N/mm)	Maximum load (N)	Pull-to-failure	Failure Mode
Specimen 1	PTF	84.7	276.9	Yes	suture cut out through tendon
Specimen 2	PTF	73.9	396.4	Yes	suture cut out through tendon
Specimen 3	PTF	107.0	385.7	Yes	suture cut out through tendon
Specimen 4	PTF	74.3	278.4	Yes	suture cut out through tendon
Specimen 5	PTF	62.0	236.6	Yes	suture cut out through tendon
Specimen 6	PTF	87.5	333.7	Yes	suture cut out through tendon
Average ± SD		81.5 ± 15.4	318.0 ± 64.6		

Stiffness, maximum load pull-to-failure, and failure mode are presented for each specimen individually. PTF, pull-to-failure; Y = yes, N = Newton, mm = millimeter

## Discussion

The most important findings of the study were that stiffness was not significantly different between the two tested techniques and that the double row technique was significantly superior to the single row technique concerning maximum load to failure. These findings confirm our hypothesis and support the use of a double row technique for re-fixation of the common flexor or extensor origin to the medial or lateral epicondyle following acute elbow dislocation.

The use of suture anchors for re-fixation of primary or secondary stabilizers of the elbow is a common and proven technique in daily clinical practice [14, 15]. Although there exists a paucity of literature concerning controlled laboratory studies evaluating different types of fixation techniques for tendon-to-bone repairs of the elbow, multiple studies already assessed the biomechanical strengths of rotator cuff repairs. Assuming the results of the shoulder to be transferable to the elbow, the ideal repair construct has to provide sufficient contact pressures at the bone-tendon-interface over the greatest possible contact area [16]. As a result, the double row repair technique has been recently evolved [16, 17]. Moreover, to avoid a functional tenodesis of the repaired tendon and thus a compromised blood supply hampering the healing, knotless and self-reinforcing repair techniques have been developed [18, 19].

The results of our controlled laboratory study support the assumption that the aforementioned findings from rotator cuff studies are valid for the elbow, too. We found the double row repair technique to be significantly stronger than the single row technique for both the common extensor and flexor origin repair. Even though reasons for this finding have not been assessed, several arguments can explain these findings. First of all, the use of an additional suture anchor has probably added further stability to the repair. Furthermore, the resultant double row construct allowed for a better distribution of the loading forces and thus were probably able to withstand significantly higher loads compared to the single row repair technique before failing [17]. Finally, a further known advantage of knotless fixation and thus a potential contributing factor to increased fixation strengths is the consistency in the fixation strengths, as previous studies have demonstrated that hand-tied knots have a high variability of strength [20, 21]. Of note, the vast majority of our constructs failed at the suture-tendon-interface with the sutures cutting out of the tendon. This mode of failure is typical for tendon-to-bone repairs and generally considered to be the weak spot of the repair [22].

Taking all of our findings into account, we believe that the double row construct is not only biomechanically but might also potentially be clinically superior to the single row construct as it allows for a reliable and early rehabilitation postoperatively. However, future comparative clinical studies have to confirm our assumption. Moreover, there exist some disadvantages of the double row repair technique in daily practice, which need to be mentioned as well. First of all, the extent of injury in case of a complex and acute elbow dislocation is most likely not limited to the flexor muscles and/or extensor muscles but also includes the primary stabilizers such as the collateral ligaments. As those might need to be re-fixated with suture anchors, too, the total amount of suture anchors used for the repair should be limited to avoid iatrogenic deterioration of the bone. Apart from that, the correct intraoperative positioning of the suture anchors gets more challenging with an increasing number of anchors used. Finally, the additional suture anchor adds surgery time and costs to the repair.

Overall, this biomechanical study provides utility by removing many external variables that may impact results, making a direct comparison of the two techniques more accurate. However, there are also inherent limitations to a cadaveric biomechanical study that cannot be controlled. The uniaxial forces applied to the common flexor or extensor tendon vertically from the humerus may not accurately reflect the dynamic loads experienced throughout a full range of motion of the elbow. More precisely, our setup did not take varus and valgus movements into account which play a substantial role in elbow dislocations. Moreover, without the contribution of healing, scarring, or muscle contractions, the measured fixation strength only simulates reconstruction immediately after surgery. Nonetheless, simulating the threshold of fixation strength immediately post-operatively may be useful information for developing appropriate rehabilitation protocols and may be useful for subsequent clinical studies.

## Conclusion

The double row technique provides significant greater stability to the re-fixated common flexor or extensor origin to the medial or lateral epicondyle. Thus, it should be considered in the development of improved repair techniques for stabilizers of the elbow.

## Abbreviations

IRB: Institutional Review Board
LCL: Lateral collateral ligament
MCL: Medial collateral ligament
N: Newton
PTF: Pull-to-failure
Y: Yes
